# Data on chemical composition of alkaloids of *Plumula nelumbinis* and antioxidant activity from thirteen habitats in China

**DOI:** 10.1016/j.dib.2018.11.004

**Published:** 2018-11-03

**Authors:** Wenyue Tian, Hui Zhi, Chao Yang, Likang Wang, Jiatang Long, Luomin Xiao, Jizheng Liang, Ying Huang, Xi Zheng, Suqing Zhao, Kun Zhang, Junxia Zheng

**Affiliations:** a Institute of Natural Medicine and Green Chemistry, School of Chemical Engineering and Light Industry, Guangdong University of Technology, Guangzhou 510006; PR China; b School of Pharmaceutical Science, Guangzhou University of Chinese Medicine, Guangzhou 510006; PR China; c Susan Lehman Cullman Laboratory for Cancer Research, Department of Chemical Biology, Ernest Mario School of Pharmacy, Rutgers, The State University of New Jersey, Piscataway, New Jersey 08854, USA; d School of Chemistry and Environment Engineering, Wuyi University, Jiangmen 529020; PR China

## Abstract

*Plumula nelumbinis* is widely consumed as tea for its pharmacological properties, which is related to its chemical composition, so the identification of the major compounds of *P. nelumbinis* is valuable. The data described in this article is supported by the research article entitled “Chemical composition of alkaloids of *Plumula nelumbinis* and their antioxidant activity from different habitats in China” (Tian et al., 2018). Included are the MS-MS Spectrograms of seven alkaloid standards and thirty alkaloids identified in the *P. nelumbinis,* which is based on ultra-performance liquid chromatography electrospray ionization quadrupole time-of-flight mass spectrometry method. Also included are the total alkaloids content and the antioxidant activity of total alkaloid in *P. nelumbinis* from 13 habitats in China, which was accomplished with three different antioxidant assays.

**Specifications table**

**Table:** 

Subject area	Chemistry, Biology
More specific subject area	Natural product chemistry
Type of data	Table and figures
How data was acquired	Ultra-performance liquid chromatography electrospray ionization quadrupole time-of-flight mass spectrometry (UPLC-ESI-QTOF-MS), UV, Tecan Infinite F200 Pro
Data format	Raw, Analyzed
Experimental factors	*Plumula nelumbinis* was extracted three times with 80% EtOH-H_2_O, dissolved in 0.1% HCl, then loaded on the pretreated D001 cation exchange resin chromatography. The sample was dissolved in MeOH and filtered through a 0.22 µm membrane filter before analysis.
Experimental features	Instrumental testing, the samples were analyzed qualitatively by UPLC-ESI-QTOF-MS.
Data source location	China
Data accessibility	The data are available with in this article
Related research article	[1] Wenyue Tian, Hui Zhi, Chao Yang, Likang Wang, Jiatang Long, Luomin Xiao, Jizheng Liang, Ying Huang, Xi Zheng, Suqing Zhao, Kun Zhang, Junxia Zheng. Chemical composition of alkaloids of *Plumula nelumbinis* and their antioxidant activity from different habitats in China [J]. **Industrial Crops and Products,** 2018, 125∶537–548.

**Value of the data**

• Method and data can be used to identify natural alkaloids by UPLC-ESI-QTOF-MS.
• The chromatographic and mass spectrometric data can be used for comparison with other studies performed on *Plumula nelumbinis*, then serve as a benchmark for other researchers to elucidate the constituents of *P. nelumbinis*.
• The alkaloid contents and the antioxidant activity data will provide a valuable reference for studies comparing the chemical and pharmacological effects of *P. nelumbinis*.

## 1 Data

**Fig. 1:**
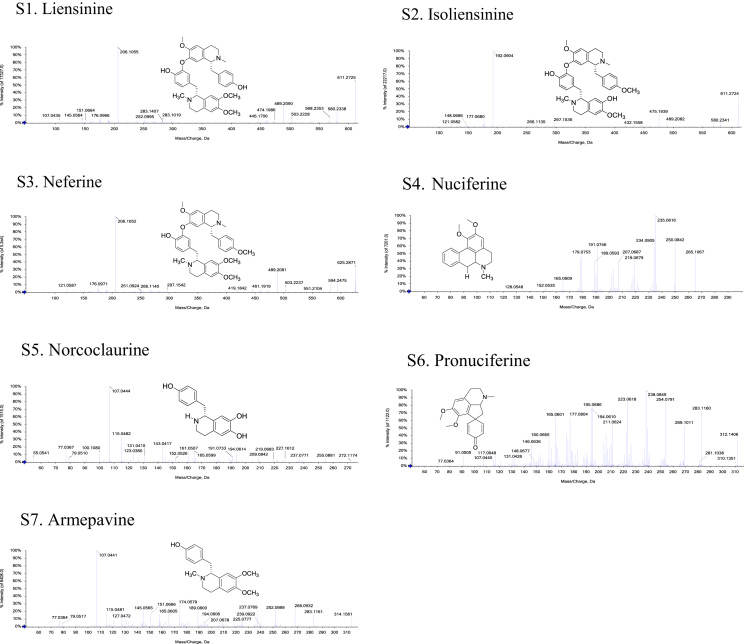
The structures and MS-MS Spectrograms of seven alkaloid standards. (S: standard).

**Fig. 2:**
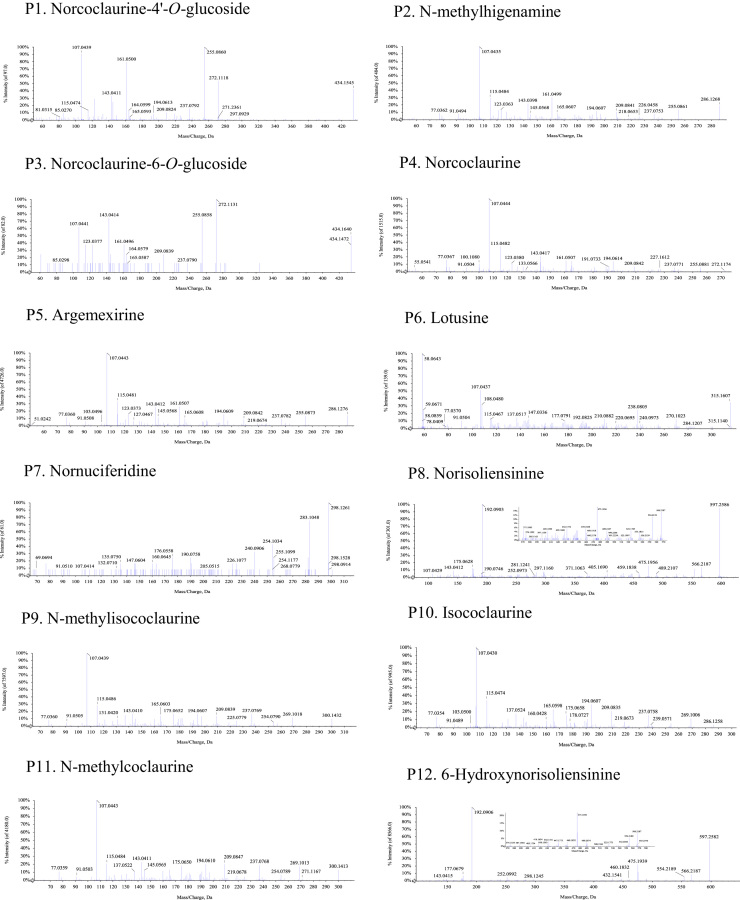

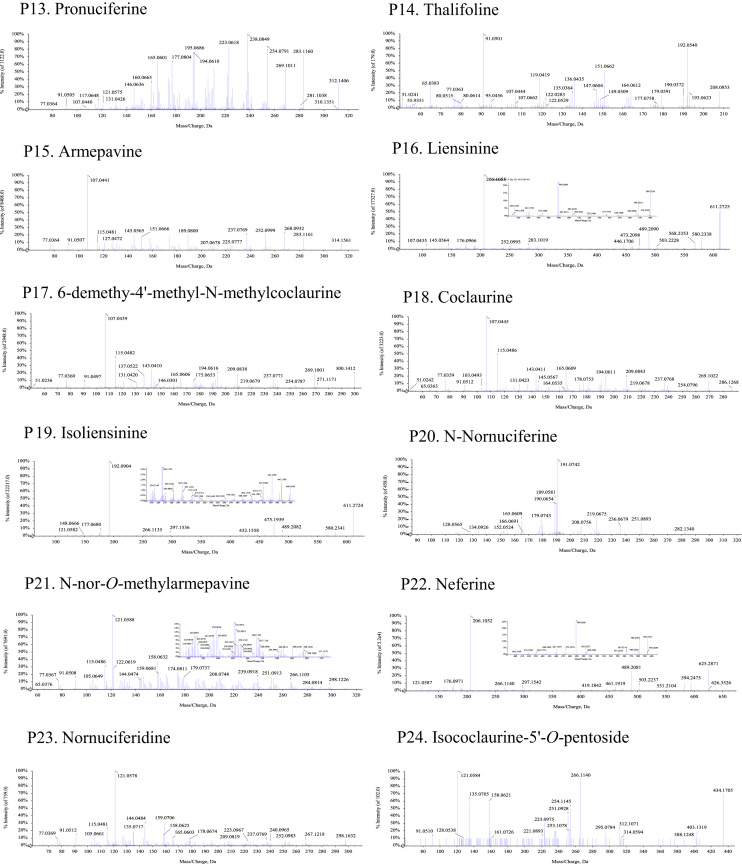

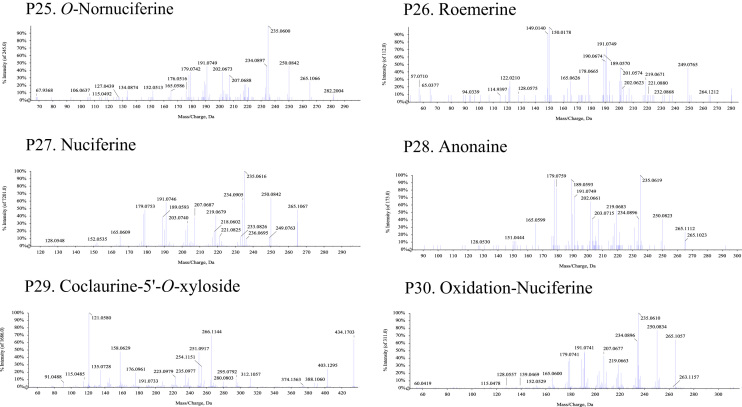
The MS-MS Spectrograms of 30 alkaloids detected in the *Plumula nelumbinis* from Xiangtan, Hunan province. (P: peak).

The structures and MS-MS spectrograms of seven alkaloid standards is provided in Fig. 1. Data in Fig. 2 presents the MS-MS spectrograms of 30 alkaloids identified in the *Plumula nelumbinis* from Xiangtan, Hunan province. Data in Table 1 includes the total alkaloids content and antioxidant capacity of *P. nelumbinis* obtained from 13 different habitats in China.

**Table 1:** Total flavonoids content and antioxidant capacity of *Plumula nelumbinis* obtained from different habitats, cited on a dry weight basis.

sample	Alkaloids (µg NE^a^/mg DW)	DPPH IC_50_ (μg/mL)	FRAP (μmol Fe(II)/mg DW)	ORAC (µmol TE^b^/mg DW^c^)
Hunanxiangtan	232.05 ± 1.18^d^	452.94 ± 2.62	0.67 ± 0.01	1.77 ± 0.002
Jiangxiguangchang	189.66 ± 0.96	116.82 ± 0.93	0.59 ± 0.03	2.21 ± 0.001
Jiangxishicheng	295.66 ± 1.18	99.85 ± 0.80	0.99 ± 0.01	1.42 ± 0.002
Shandongweihu	177.31 ± 0.27	469.68 ± 1.76	0.70 ± 0.02	4.60 ± 0.003
Shandongheze	286.98 ± 4.05	482.25 ± 2.86	0.94 ± 0.03	1.42 ± 0.001
Zhejianghangzhou	463.49 ± 1.34	217.29 ± 1.74	1.27 ± 0.03	2.95 ± 0.002
Fujiannanping	175.04 ± 0.81	156.78 ± 1.25	0.44 ± 0.01	0.30 ± 0.004
Shanxiweinan	126.32 ± 2.01	>1000	0.43 ± 0.04	0.25 ± 0.003
Hubeihonghu	119.84 ± 0.11	512.69 ± 3.10	0.44 ± 0.04	0.42 ± 0.004
Anhuibozhou	222.75 ± 0.27	81.86 ± 0.65	1.50 ± 0.05	5.53 ± 0.003
Yunnanwenshan	541.54 ± 1.88	93.99 ± 0.75	0.96 ± 0.00	4.19 ± 0.006
Zhejiangwuyi	128.48 ± 0.61	>1000	0.94 ± 0.00	2.81 ± 0.005
Fujianfuzhou	235.36 ± 0.76	84.98 ± 0.68	0.95 ± 0.01	0.69 ± 0.008

a NE is the abbreviation of Neferine equivalents,

b TE is the abbreviation of Trolox equivalents,

c DW is the abbreviation of dry weight of sample;

d Each value is expressed as mean ± SD (N=3).

## 2 Experimental design, materials, and methods

### 2.1 Chemicals and materials

*The P. nelumbinis* samples were collected from 13 different habitats in China, during August, 2017. Nuciferine, pronuciferine, liensinine, isoliensinine, neferine, armepavine and norcoclaurine were obtained from Junmu Biotechnology Co. Ltd (Guangzhou, China). 2,2׳-Azobis(2-amidinopropane dihydrochloride), 6-hydroxy-2,5,7,8-tetramethylchroman-2-carboxylic acid, L-ascorbic acid, sodium fluorescein, 2,4,6-tri(2-pyridyl)-s-triazine and 2,2-diphenyl-1-picrylhydrazyl were purchased from the Sigma-Aldrich Chemical Co. Ltd (Saint Louis, MO, USA). Methanol, acetonitrile (all chromatographic grade) were purchased from Swedish Oceanpak Co. And the formic acid (chromatographic grade) were purchased from the Fine Chemical Co. Ltd (Tianjin, PR China). Ultrapure water was purified using a Milli-Q Advantage A10 system (Millipore, Billerica, MA, USA). And the detailed description of chemicals and materials has been described in Ref. [1].

### 2.2 Extraction and isolation

Dried *P. nelumbinis* was extracted three time with 80% EtOH-H_2_O, and the ethonal extract isolated by D001 cation exchange resin chromatography, as washed with water and 95% ethanol (included 1% ammonia) in turn, the 95% ethanol/ammonia wash fraction contains the bulk of the alkaloids, which was stored at −20°C after freeze-drying. The detail procedures were described in Ref. [1].

### 2.3 UPLC-ESI-QTOF-MS parameter

Identification of the samples were carried out using a Shimadzu Prominence UPLC system (Nexera UHPLC LC-30A, Kyoto, Japan) equipped with a A triple TOF^TM^ 5600^+^ mass spectrometer (AB Sciex, Foster City, CA). And the detail parameter setting of LC system and mass spectrometric detection were selected as described in Ref. [1]. Briefly, the analysis was carried out using a Waters Acquity UPLC BEH C_18_ column (2.1 mm × 100 mm, 1.7 µm) and the mobile phase of acetonitrile (containing 0.1% formic acid)-water (containing 0.1% formic acid) at a flow rate of 0.3 mL/min with the column temperature kept at 40 °C and the injection volume of 3 µL. And the mass spectrometric detector was operated in the positive ESI mode and was equipped with a DuoSpray^TM^ source (AB Sciex, Foster City, CA).

### 2.4 Total alkaloid content

The total alkaloid content of *P. nelumbinis* was determined using the acid dye colorimetric method according to the methods in Ref. [1]. In which, neferine was used as a reference compound.

### 2.5 Antioxidant activity of total alkaloids

In this study, three different chemical methods, like the DPPH, ORAC and FRAP assays were used to evaluated the antioxidant activity of the total alkaloids which from different habitats.

The free radical-scavenging activity of the total alkaloids of *P. nelumbinis* was determined with a DPPH test according to the methods in Ref. [1].

The ORAC assay was completed in accordance with the previously described according to the methods in Ref. [1].

The Ferric reducing antioxidant power assay of *P. nelumbinis* were determined according to the methods in Ref. [1].
